# Simultaneous kidney–islet transplantation from a donor after circulatory death using normothermic regional perfusion

**DOI:** 10.3389/ti.2026.16830

**Published:** 2026-07-09

**Authors:** Laure Esposito, Orianne Villard, Thibault Austry, Christophe Broca, John Devos, Guillaume Ducos, Diane Osinski, Nelly Puech, Cedric Idri, Thibault Boisroux, Stéphanie Ruiz, Chloe Medrano, Thomas Prudhomme, Mokrane Fatima, Nassim Kamar

**Affiliations:** 1 Department of Nephrology and Organ Transplantation, Toulouse University Hospital, Toulouse, France; 2 Department of Endocrinology and Diabetes, Montpellier University Hospital, Institute of Functional Genomics, CNRS, INSERM, University of Montpellier, Montpellier, France; 3 Department of Radiology, Toulouse University Hospital, Toulouse, France; 4 Department of Cell and Tissue Engineering, Laboratory of Cell Therapy for Diabetes, Montpellier University Hospital, University Montpellier, Montpellier, France; 5 Coordination des Prélèvements d’Organes et de Tissus, Toulouse University Hospital, Toulouse, France; 6 Department of Diabetes, Toulouse University Hospital, Toulouse, France; 7 Department of Urology, Andrology and Kidney transplantation,Toulouse University Hospital, Toulouse, France; 8 Department of Vascular Surgery, Toulouse University Hospital, Toulouse, France; 9 Department of Anesthesiology and Intensive Care, Toulouse University Hospital, Toulouse, France; 10 Université de Toulouse, Toulouse, France; 11 Institute of Metabolic and Cardiovascular Diseases (I2MC), Institut National de la Santé et de la Recherche Médicale (INSERM) U1297, Toulouse, France

**Keywords:** glycemic control, islets, isolation, kidney transplantation, Maastricht III

Dear Editors,

Patients with insulin-dependent diabetes and advanced chronic kidney disease frequently require β-cell replacement therapy, either at the time of kidney transplantation or as a staged procedure thereafter. Two established strategies for β-cell replacement include whole-organ pancreas transplantation and pancreatic islet transplantation. Both approaches can be performed either simultaneously with kidney transplantation or sequentially. Simultaneous pancreas–kidney transplantation (SPK) remains the gold standard for individuals with type 1 diabetes and end-stage kidney disease. However, a substantial proportion of candidates are unsuitable due to prohibitive perioperative risks. In such cases, simultaneous islet–kidney transplantation (SIK) may be considered. The procedural risk associated with intraportal infusion of isolated pancreatic islets is lower compared to whole pancreas transplantation. While SPK achieves higher rates of insulin independence, SIK has demonstrated quite comparable benefits in terms of glycemic stability, prevention of severe hypoglycemia, patient survival, cardiovascular outcomes, and kidney allograft survival—alongside a markedly reduced burden of surgical and metabolic complications [1–3]. However, donors’ and recipients’ characteristics can impact the results of islet transplantation.

SPK and SIK were initially performed using organs and islets from donors after brain death (DBD). More recently, kidney, pancreas, islet-alone, and islet-after-kidney transplantations from controlled donors after circulatory death (DCD; Maastricht category III donors) have also been utilized [4, 5].

Herein, we report the first SIK performed in France using a kidney and islets from a controlled DCD in whom, according to the national French protocol, normothermic regional perfusion (NRP) was implemented to restore *in situ* circulation of abdominal organs [6].

A 45-year-old non-Human leukocyte antigen sensitized man (weight 53 kg) with a history of complicated, longstanding type 1 diabetes (onset at age 20) and on hemodialysis for 10 years was considered for SIK transplantation due to suboptimal continuous glucose monitoring metrics [HbA1c 8%; time in range (70–180 mg/dL) 36%; time <70 mg/dL 20%, including 5% < 54 mg/dL with hypoglycemia unawareness; and high glycemic variability (coefficient of variation 40%; target <36%)]. Due to extensive comorbidities, primarily ischemic cardiomyopathy and peripheral arterial disease, whole pancreas transplantation was contraindicated.

The donor was a 60-year-old man (BMI 27.5 kg/m^2^, weight 66 kg) in whom life-sustaining therapy was withdrawn following a large ischemic stroke without neurological recovery after 4 days in the ICU. During procurement, the functional warm ischemia time was 28 min (2 min from mean blood pressure < 50 mmHg to circulatory arrest, followed by 26 min of asystole, including the required 5-min no-touch interval). Cannulation and radiologic verification required 21 min. Peak transaminases during NRP were twice the upper limit of normal (ULN), and lipase decreased from 3× to 2.5× ULN during NRP, which lasted 3.5 h.

Immediately after procurement, the kidney was preserved on a hypothermic perfusion machine (Organ Recovery system) without oxygenation. The cold ischemia time was 21.8 h, and the warm ischemia time (Time between withdrawal from machine perfusion and renal reperfusion) was 66 min. Kidney transplantation was performed by both urologists and vascular surgeons, as an iliofemoral bypass was required prior to graft implantation. The postoperative course was uneventful, with immediate recovery of urine output and graft function. Serum creatinine decreased from 490 μmol/L pre-transplantation to 108 μmol/L at month 3 (Figure 1A).

**FIGURE 1:**
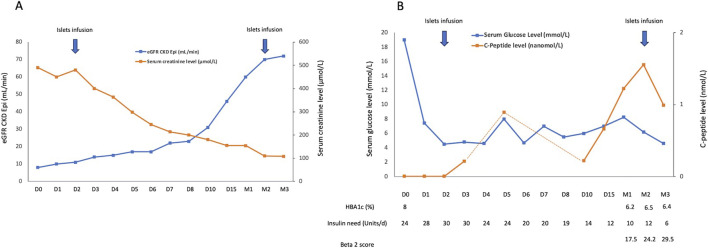
Outcome of kidney function **(A)** and glycemic parameters **(B)** after simultaneous islet kidney transplantation.

The patient received induction therapy with anti-thymocyte globulins (Thymoglobulin®, 1.25 mg/kg/day for 4 days), followed by maintenance immunosuppression including tacrolimus (target trough level 8–12 ng/mL), mycophenolic acid (1 g twice daily), and corticosteroids (500 mg on day 0, 250 mg on day 1, then 20 mg/day until day 7, when discontinued).

The pancreas was sent to the Cell Therapy Unit at Montpellier University Hospital for islet isolation, which was completed in 5 h. The pancreas cold ischemia time was 5.5 h. Enzymatic digestion and continuous density gradient separation yielded 103,800 islet equivalents (IEQ; 1958 IEQ/kg), with 89% viability and 48% purity, meeting criteria for clinical transplantation of islets alone, simultaneously and after kidney. Islets were cultured for 24 h. Two days after organ procurement and kidney transplantation, the islets were prepared, packaged, and transported from Montpellier University Hospital to Toulouse University Hospital, where intraportal infusion was performed radiologically via percutaneous portal vein catheterization. Continuous intravenous insulin was administered to maintain strict euglycemia (target 0.8–1.2 g/L) during early engraftment. Anticoagulation included 70 IU/kg unfractionated heparin during infusion, followed by systemic heparin for 48 h (activated partial thromboplastin time 1.5–2× control), then prophylactic anticoagulation until day 7.

Endogenous insulin secretion became detectable within 24 h, with C-peptide levels increasing from 0 to 0.64 ng/mL on day 1 and reaching 4.7 ng/mL at month 2, consistent with effective engraftment (Figure 1B). Over the subsequent 2 months, mean glucose was 9.35 mmol/L; time in range (70–180 mg/dL) improved to 56%, time in hypoglycemia (<70 mg/dL) decreased to 3%, time <54 mg/dL to 0%, and no severe hypoglycemia occurred. At month 2, he received a second islets infusion (244,099 IEQ) obtained from DBD. Daily insulin requirements and glycated hemoglobin decreased over time (Figure 1B).

Islet isolation from DBD pancreases generally yields higher β-cell quantities and, consequently, higher transplantation eligibility rates compared to controlled DCD, likely due to the initial period of warm ischemia [7]. However, in most studies, controlled DCD donors did not benefit from NRP, which is mandatory in France and not routinely implemented in many countries. Nevertheless, it was previously shown that abdominal NRP after controlled DCD improves pancreatic islet isolation yield {Doppenberg, 2025 #2883}. In the present, although the IEQ infused the first time was relatively low, it allowed to improve the glycemic control and the later was again improved by the second infusion. Both *in vitro* and clinical studies have demonstrated that when a sufficient number of islets is infused, viability and function are comparable between controlled DCD and DBD sources, resulting in similar insulin secretion and glycemic control [7].

In summary, this case report confirms that SIK using organs from DCD donors is feasible and effective, potentially expanding access to β-cell replacement therapy for patients with insulin-dependent diabetes and advanced chronic kidney disease. The use of NRP may improve β-cell yield from donor pancreases following circulatory death.

## Data Availability

The raw data supporting the conclusions of this article will be made available by the authors, without undue reservation.
